# Assessment of Intratumoral and Peritumoral Computed Tomography Radiomics for Predicting Pathological Complete Response to Neoadjuvant Chemoradiation in Patients With Esophageal Squamous Cell Carcinoma

**DOI:** 10.1001/jamanetworkopen.2020.15927

**Published:** 2020-09-10

**Authors:** Yihuai Hu, Chenyi Xie, Hong Yang, Joshua W. K. Ho, Jing Wen, Lujun Han, Keith W. H. Chiu, Jianhua Fu, Varut Vardhanabhuti

**Affiliations:** 1 Department of Thoracic Surgery, Sun Yat-sen University Cancer Center, Guangzhou, China; 2 State Key Laboratory of Oncology in South China, Collaborative Innovation Center for Cancer Medicine, Guangzhou, China; 3 Guangdong Esophageal Cancer Institute, Guangzhou, China; 4 Department of Diagnostic Radiology, Li Ka Shing Faculty of Medicine, University of Hong Kong, Hong Kong SAR, China; 5 School of Biomedical Sciences, Li Ka Shing Faculty of Medicine, University of Hong Kong, Hong Kong SAR, China; 6 Department of Medical Imaging, Sun Yat-sen University Cancer Center, Guangzhou, China

## Abstract

**Importance:**

For patients with locally advanced esophageal squamous cell carcinoma, neoadjuvant chemoradiation has been shown to improve long-term outcomes, but the treatment response varies among patients. Accurate pretreatment prediction of response remains an urgent need.

**Objective:**

To determine whether peritumoral radiomics features derived from baseline computed tomography images could provide valuable information about neoadjuvant chemoradiation response and enhance the ability of intratumoral radiomics to estimate pathological complete response.

**Design, Setting, and Participants:**

A total of 231 patients with esophageal squamous cell carcinoma, who underwent baseline contrast-enhanced computed tomography and received neoadjuvant chemoradiation followed by surgery at 2 institutions in China, were consecutively included. This diagnostic study used single-institution data between April 2007 and December 2018 to extract radiomics features from intratumoral and peritumoral regions and established intratumoral, peritumoral, and combined radiomics models using different classifiers. External validation was conducted using independent data collected from another hospital during the same period. Radiogenomics analysis using gene expression profile was done in a subgroup of the training set for pathophysiological explanation. Data were analyzed from June to December 2019.

**Exposures:**

Computed tomography–based radiomics.

**Main Outcomes and Measures:**

The discriminative performances of radiomics models were measured by area under the receiver operating characteristic curve.

**Results:**

Among the 231 patients included (192 men [83.1%]; mean [SD] age, 59.8 [8.7] years), the optimal intratumoral and peritumoral radiomics models yielded similar areas under the receiver operating characteristic curve of 0.730 (95% CI, 0.609-0.850) and 0.734 (0.613-0.854), respectively. The combined model was composed of 7 intratumoral and 6 peritumoral features and achieved better discriminative performance, with an area under the receiver operating characteristic curve of 0.852 (95% CI, 0.753-0.951), accuracy of 84.3%, sensitivity of 90.3%, and specificity of 79.5% in the test set. Gene sets associated with the combined model mainly involved lymphocyte-mediated immunity. The association of peritumoral area with response identification might be partially attributed to type I interferon–related biological process.

**Conclusions and Relevance:**

A combination of peritumoral radiomics features appears to improve the predictive performance of intratumoral radiomics to estimate pathological complete response after neoadjuvant chemoradiation in patients with esophageal squamous cell carcinoma. This study underlines the significant application of peritumoral radiomics to assess treatment response in clinical practice.

## Key Points

**Question:**

Are peritumoral radiomics features extracted from pretreatment computed tomography images predictive of pathological complete response following neoadjuvant chemoradiation in patients with esophageal squamous cell carcinoma?

**Findings:**

In this diagnostic study of 231 patients, the developed model integrating intratumoral and peritumoral radiomics features achieved improvement of predictive performance (area under the receiver operating characteristic curve, 0.852) compared with the conventionally constructed model merely using intratumoral radiomics features (area under the receiver operating characteristic curve, 0.730).

**Meaning:**

Peritumoral radiomics may provide additional predictive value for treatment response estimation in esophageal squamous cell carcinoma and thus benefit individualized therapeutic strategies.

## Teaser

This diagnostic study assesses the use of intratumoral and peritumoral computed tomography (CT) radiomics for predicting pathological complete response to neoadjuvant chemoradiation in patients with esophageal squamous cell carcinoma (ESCC).

## Introduction

Esophageal cancer (EC) is one of the most fatal malignant neoplasms worldwide.^1^ In Asia, more than 90% of patients with EC receive a diagnosis of esophageal squamous cell carcinoma (ESCC), compared with approximately 20% of patients with EC in Western countries.^2^ Neoadjuvant chemoradiotherapy (nCRT) has been confirmed by large-scale randomized clinical trials to benefit tumor resectability and long-term survival of patients with locally advanced ESCC.^3,4,5,6^ However, treatment response patterns vary from patient to patient. Pathological complete response (pCR) has been considered as a strong prognostic factor for favorable outcome.^7,8,9^ Approximately less than one-half of patients receiving nCRT have been shown to achieve pCR.^3,4,10^ Patients with limited tumor regression are likely to experience unnecessary adverse events and run the risk of delay of operation and even disease progression. Therefore, accurate estimation of nCRT response is meaningful for pretherapeutic decision-making. A practical and noninvasive approach to precisely assess therapeutic response before treatment implementation is required.

Previous studies^11,12,13,14,15,16^ have evaluated the capability of conventional parameters derived from computed tomography (CT) or integrated positron emission tomography to estimate treatment response. In these studies, most examinations were performed in the early course of or after nCRT, which is of little importance regarding the initial planning of therapy regimen. The use of conventional parameters from baseline imaging alone has limited value in pretreatment prediction.^17,18^ Radiomics has provided novel information from medical images.^19^ It has been reported that radiomics features of the core tumoral area present significant predictive power for the treatment response in EC.^20,21,22,23^ Recently, radiomics features derived from the peritumoral area have also been shown to be predictive in response assessment in other cancers. Braman et al^24^ showed that peritumoral radiomics possessed valuable pCR-related attributes in breast cancer across different molecular types. Sun and colleagues^25^ reported an area under the receiver operating characteristic curve (AUC) of 0.999 in the testing set of a magnetic resonance imaging–based model combining intratumoral and peritumoral radiomics for predicting response to neoadjuvant chemotherapy in cervical cancer. Khorrami et al^26^ reported that peritumoral radiomics features derived from CT images were predictive of response to chemotherapy in lung adenocarcinoma.

Previous radiomics studies^20,21,22,23,27^ in EC mainly focused on the intratumoral region alone, whereas little is known about the role of peritumoral radiomics features, which are likely to provide crucial but easily overlooked information about pCR. In this context, we hypothesized that the subtle structural deformations occurring around esophageal wall regions seen on CT images could be potential biomarkers to nCRT response in ESCC. Thus, we aimed to develop baseline CT-based models to identify pCR using intratumoral and peritumoral radiomics features separately and in combination and to validate their performances in an independent cohort. Furthermore, we also performed a radiogenomics analysis to investigate the pathophysiological features associated with radiomics signatures and how peritumoral features contributed to the predictive ability.

## Methods

### Patients

**Figure 1.**
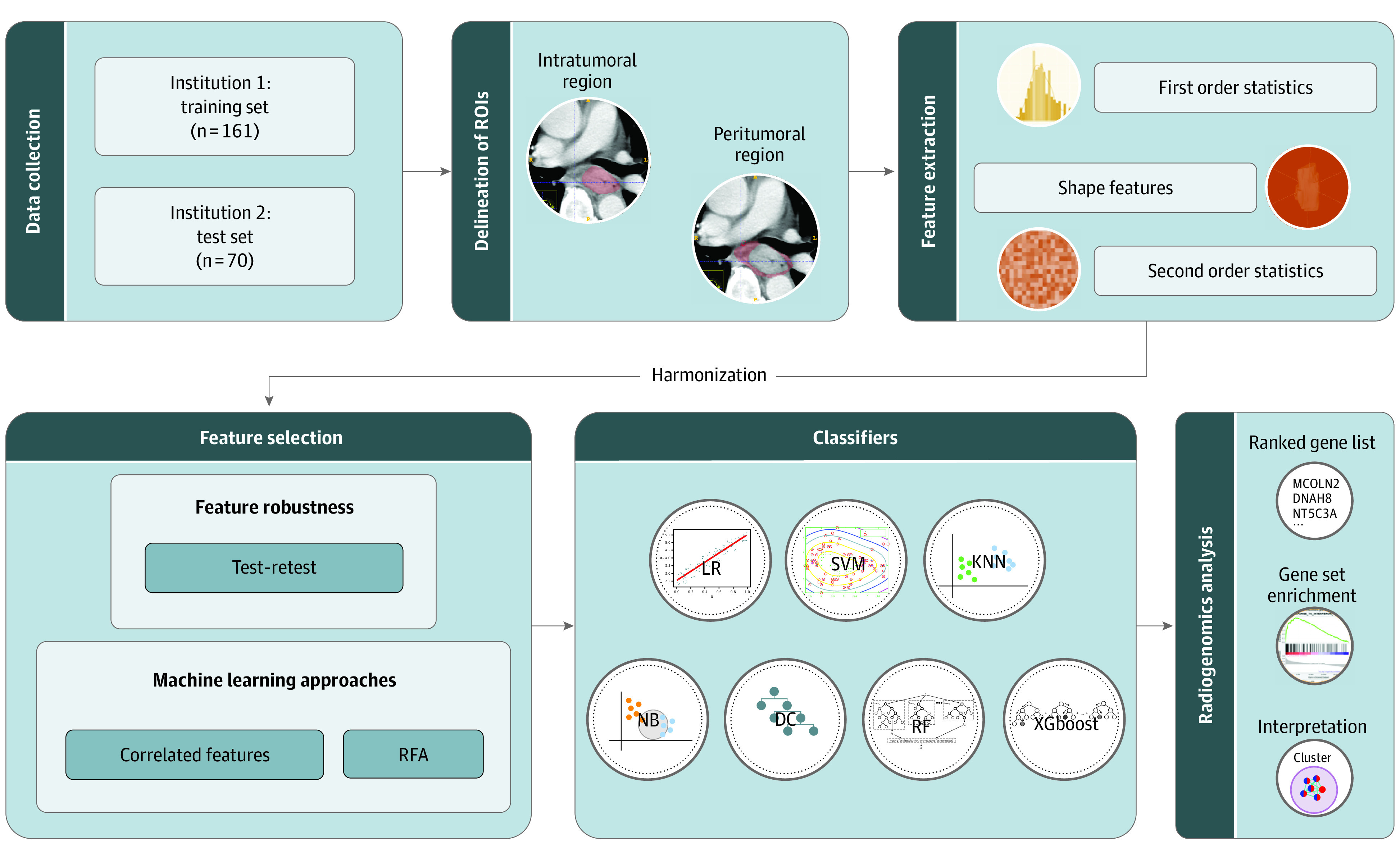
Analysis Flowchart DC indicates decision tree; KNN, k-nearest neighbors; LR, linear regression; NB, naive bayes; RF, random forest; RFA, recursive feature addition; ROI, region of interest; SVM, support vector machine; and XGboost, extreme gradient boosting.

This diagnostic study was approved by the institutional review boards of Sun Yat-sen University Cancer Center and University of Hong Kong. The requirement for informed consent was waived because the study was deemed to pose no additional risk to patients and the data were deidentified. The study follows the Standards for Reporting of Diagnostic Accuracy (STARD) reporting guideline. Figure 1 shows the experimental design.

We retrieved records between April 2007 and December 2018 from Sun Yat-sen University Cancer Center (institution 1) and University of Hong Kong (institution 2). The flow of patient selection is depicted in eFigure 1 in the Supplement. Images from patients at institution 1 were used as the training set, and those from patients at institution 2 were used as the test set. Enrolled patients with ESCC underwent pretreatment contrast-enhanced CT scans, with coverage from the neck to the upper abdomen, and then underwent nCRT followed by radical esophagectomy. All patients received platinum-based chemotherapy and concurrent radiotherapy preoperatively (see eAppendix 1 in the Supplement for detailed regimens). Radical esophagectomy was performed 4 to 8 weeks after the completion of nCRT. Pathological response was assessed by pathologists specialized in EC, and pCR was identified as no presence of viable cancer cells in all the resected specimens.

### Delineation of Regions of Interest

CT image acquisition is detailed in eAppendix 1 in the Supplement. The pretreatment contrast-enhanced CT images were retrieved from the picture archiving and communication system. Two senior radiologists (V.V. and L.H.) with 10 and 9 years of experience, respectively, were blinded to pathological response and manually delineated the intratumoral and peritumoral regions of interest using ITK-SNAP image segmentation software version 3.6 (Yushkevich P and Gerig G). The intratumoral delineation covered the whole tumor in all slices with the primary lesion present. The peritumoral region was empirically annotated by radiologists including the adjacent tissue and lymph nodes immediately around the esophagus, where the airway, aorta, vertebrae, and azygos vein were excluded. We further conducted a test-retest study using a randomly selected subset of 30 patients from the training set. Features extracted from 2 sets of regions of interest and contoured separately by 2 radiologists in a blinded fashion were used to calculate the intraclass correlation coefficients. Features with intraclass correlation coefficients greater than 0.8 were regarded as robust features with good reproducibility and were selected for further analysis (1208 of 1316 of intratumoral features and 1036 of 1316 of the peritumoral features had intraclass correlation coefficients >0.80).

### Feature Extraction, Preprocessing, and Selection

Radiomics features were extracted using PyRadiomics image extraction software version 3.0.^28^ Defined features from original, wavelet-filtered, and Laplacian of Gaussian–filtered images were extracted. Additional details of feature extraction and definition are specified in eAppendix 1 in the Supplement. Radiomics features collected from different institutions were first harmonized using ComBat^29^ to minimize the batch effect caused by differences in CT acquisition and reconstruction parameters. To remove potentially redundant features and decrease data dimensions, we conducted feature selection in the training set. First, correlated features were grouped by Pearson correlation coefficient (>0.80), and the most predictive ones were retained. Correlated features in each group were fitted into a decision tree model and ranked according to their contribution for the prediction of pCR status. The top feature was selected as the representative of this group and retained. Second, we adopted a wrapper method using recursive feature addition algorithm to find the most predictable features. Features were ranked according to their relevant importance, and contributable ones were added in a recursive process using the corresponding classifiers.

### Classifiers

The selected features were used to train prediction models. Multiple classifiers that were frequently used in radiomics studies were adopted to achieve the best predictive performance. Classifiers tested in this study included linear regression, support vector machine (SVM) with linear kernel, SVM with radial basis function kernel, k-nearest neighbors, naive bayes, decision tree, random forest, and extreme gradient boosting. The optimal classifier with the best AUC was used for further exploration. The classification probability was regarded as the radiomics score.

### Radiogenomics Analysis

To explore the association of radiomics signatures and corresponding pathophysiological features, we used the RNA sequencing data to establish links to transcriptome level. Procedures of RNA-seq are shown in eAppendix 1 in the Supplement. Gene expression level was normalized by calculating fragments per kilobase of exon per million fragments. We calculated Spearman rank correlation coefficients between expression levels and radiomics scores. A total of 24 860 genes were ranked by coefficients and input to perform gene set enrichment analysis using the gene set enrichment analysis reranked tool (eAppendix 2 in the Supplement).^30^ The gene set collection of Gene Ontology Biological Process from the Molecular Signatures Database^31^ was tested. The result of enrichment analysis was then demonstrated using the EnrichementMap^32^ on Cytoscape^33^ software version 3.8.0 to create an enrichment map of enriched gene sets with *Q* values less than 0.05. Clustering was done using the AutoAnnotate tool^34^ version 1.3.3 and then manually modified and labeled.

### Statistical Analysis

The statistical differences were calculated by χ^2^ or Fisher exact test for categorical variables and the Kruskal-Wallis test for numeric variables. The performances of radiomics models were quantified by the AUC and *P* values for differences were calculated by the Delong test.^35^ The cutoff points of accuracy, sensitivity, and specificity were determined by the Youden Index. Calibration curve and decision curve were performed to test the calibration performance and clinical utility.^36^ Univariable analysis was used to detect the correlations of clinical parameters for pCR. The influence of clinical parameters on the performance was assessed by stratification analysis. A 2-tailed *P* < .05 was regarded as statistically significant. Python software version 3.7 (Python) was used for graphic depiction, and R statistical software version 3.3.3 (R Project for Statistical Computing) was used for statistical analysis (eAppendix 1 in the Supplement). Data were analyzed from June to December 2019.

## Results

### Baseline Characteristics

A total of 231 patients were enrolled in this study, including 161 in the training set and 70 in the external test set (Table 1). The mean (SD) age of all patients was 59.8 (8.7) years; 192 (83.1%) were men, and 39 (16.9%) were women. Patients with stage III disease accounted for the majority (173 patients [74.9%]). The pCR rates of the training and test sets were 46.0% and 44.3%, respectively.

**Table 1.** Patient Characteristics for Training and Test Sets

Characteristic	Patients, No. (%)					
	Institution 1 (training set)			Institution 2 (test set)		
	Non-pCR (n = 87)	pCR (n = 74)	*P* value	Non-pCR (n = 39)	pCR (n = 31)	*P* value
Age, mean (SD), y	58.26 (6.77)	57.58 (7.00)	.53	64.85 (8.83)	63.26 (12.67)	.54
Sex						
Male	72 (83)	62 (84)	>.99	33 (85)	25 (81)	.91
Female	15 (17)	12 (16)		6 (15)	6 (19)	
Clinical T stage^a^						
1b	0	1 (1)	.59	0	0	.50
2	23 (26)	19 (26)		1 (23)	2 (7)	
3	61 (70)	53 (72)		37 (95)	29 (93)	
4a	3 (4)	1 (1)		1 (2)	0	
Clinical N stage^a^						
0	3 (3)	5 (7)	.43	3 (8)	2 (7)	.51
1	40 (46)	39 (53)		12 (31)	15 (48)	
2	37 (43)	23 (31)		20 (51)	12 (39)	
3	7 (8)	7 (9)		4 (10)	2 (6)	
Clinical stage group						
I	0	1 (1)	.69	0	0	.65
II	13 (15)	13 (18)		3 (8)	2 (7)	
III	64 (74)	51 (69)		31 (80)	27 (87)	
IV A	10 (11)	9 (12)		5 (12)	2 (6)	
Tumor location						
Proximal third	6 (7)	11 (15)	.23	2 (5)	0	.38
Middle third	52 (60)	43 (58)		18 (46)	13 (42)	
Distal third	29 (33)	20 (27)		19 (49)	18 (58)	
Histologic grade						
G1	5 (6)	3 (4)	.38	0	3 (10)	.10
G2	52 (60)	52 (70)		30 (77)	19 (61)	
G3	30 (34)	19 (26)		9 (23)	9 (29)	
Tobacco use						
No	33 (38)	30 (41)	.86	13 (33)	11 (36)	>.99
Yes	54 (62)	44 (59)		26 (67)	20 (64)	
Alcohol use						
No	56 (64)	53 (72)	.42	15 (39)	15 (48)	.56
Yes	31 (36)	21 (28)		24 (61)	16 (52)	
Family history of cancer						
No	69 (79)	57 (77)	.87	33 (85)	26 (84)	>.99
Yes	18 (21)	17 (23)		6 (15)	5 (16)	

Abbreviation: pCR, pathological complete response.

^a^ Cancer staging was done with the American Joint Committee on Cancer TNM staging system (8th edition).

### Intratumoral and Peritumoral Radiomics Models

We used 8 classifiers to construct radiomics models with intratumoral or peritumoral features. Comparisons of performance across different classifiers are shown in eTable 1 in the Supplement. The intratumoral model achieved the highest AUC of 0.730 (95% CI, 0.609-0.850) for the test set by using the SVM with radial basis function kernel classifier, whereas the peritumoral model performed best in extreme gradient boosting with an AUC of 0.734 (0.613-0.854). Intriguingly, these 2 models demonstrated similar performance, indicating that peritumoral area did possess radiomics properties relevant to treatment response. The 16 intratumoral features and 8 peritumoral features that constituted the optimal models are listed in eTable 2 in the Supplement. Because the wrapped feature selection method was classifier specific, the number for selected features varied for different classifiers (eTable 3 in the Supplement).

### Combined Radiomics Model

**Figure 2.**
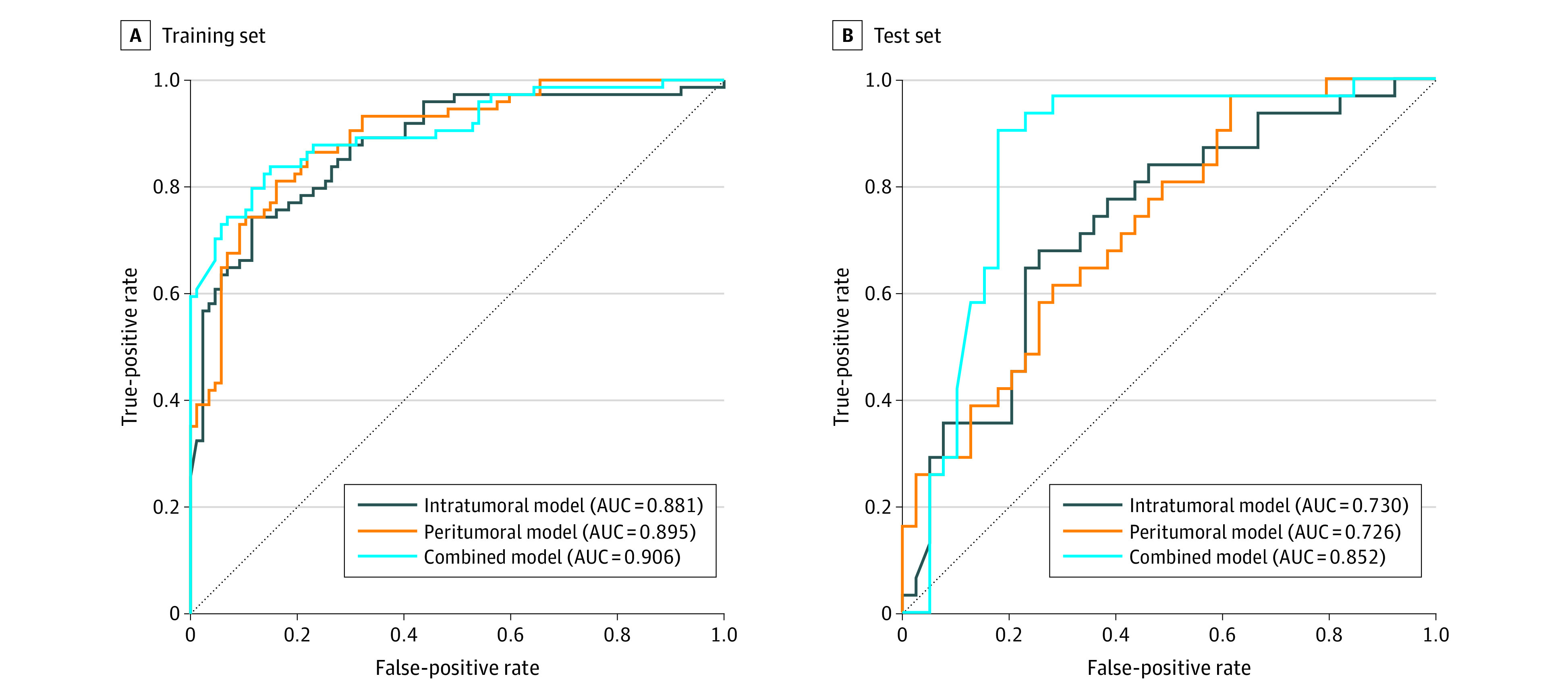
Predictive Performance of Radiomics Models Graphs show receiver operating characteristic curve curves of the intratumoral, peritumoral, and combined radiomics models constructed by support vector machine with radial basis function kernel for training set (A) and test set (B). AUC indicates area under the receiver operating characteristic curve.

**Figure 3.**
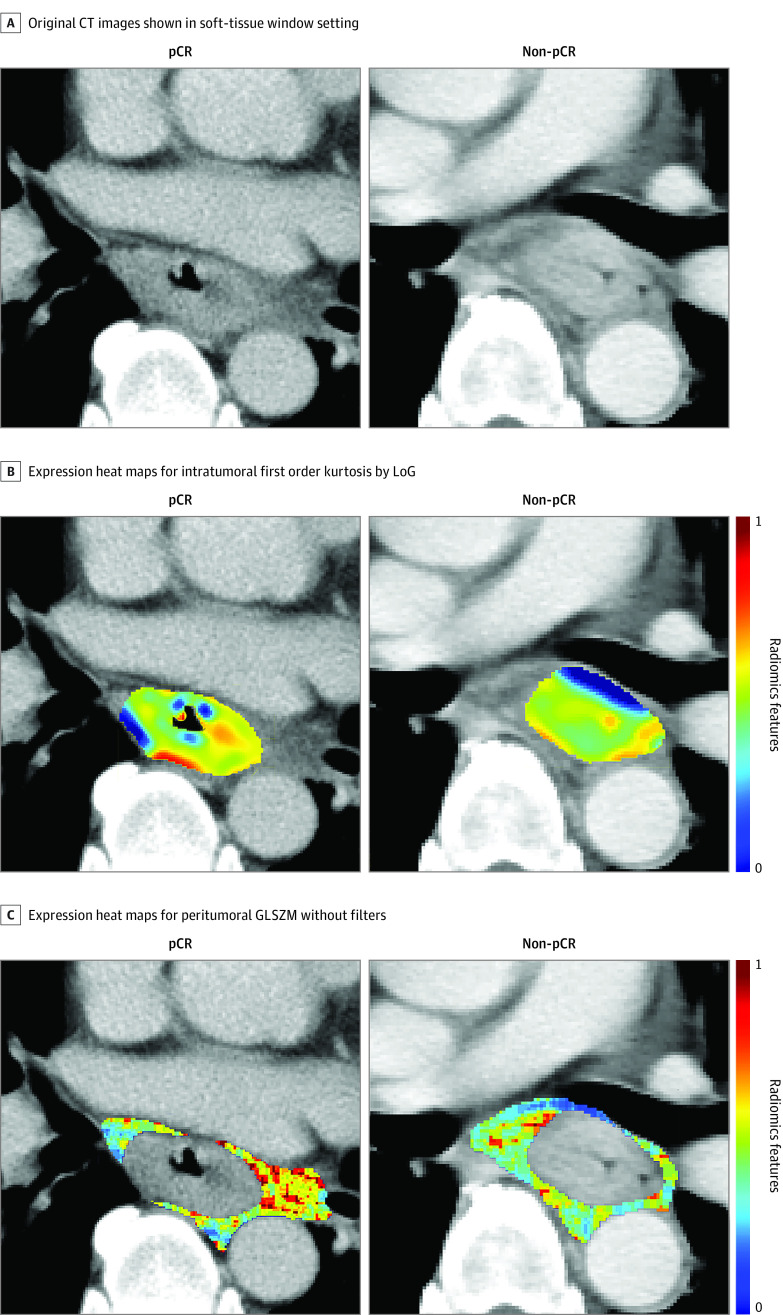
Radiomics Feature Maps Radiologist-annotated intratumoral and peritumoral regions and the corresponding radiomics expression heatmaps for top selected feature on representative CT images from patients with and without pathological complete response (pCR). Panel A shows original computed tomography (CT) images in the soft-tissue window setting. Panel B shows expression heat maps for intratumoral first order kurtosis by Laplacian of Gaussian (LoG; σ = 2 mm). Panel C shows expression heat maps for peritumoral gray level nonuniformity normalized (GLSZM) without filters. Radiomics features were scaled between 0 and 1 for comparison. Red and blue correspond to higher and lower values, respectively.

Performance was significantly improved by combining intratumoral and peritumoral features for all 8 classifiers (eTable 1 in the Supplement). The optimal performance was achieved by using SVM with radial basis function kernel with an AUC of 0.852 (95% CI, 0.753-0.951) and showed a significant improvement compared with the intratumoral model (AUC, 0.881; 95% CI, 0.827-0.835; *P* = .047) (Figure 2). The model showed good predictive value, with an accuracy of 83.9%, sensitivity of 82.4%, specificity of 85.1%, positive predictive value of 82.4%, and negative predictive value of 85.1% in the training set, and an accuracy of 84.3%, sensitivity of 90.3%, specificity of 79.5%, positive predictive value of 77.8%, and negative predictive value of 91.2% in the test set. This model was composed of 7 intratumoral and 6 peritumoral features (Table 2). One feature from original images, 7 from wavelet-filtered images, and 6 from Laplacian of Gaussian–filtered images were selected. Basic metrics first-order statistics (median and kurtosis) and high-dimensional textual features (gray level co-occurrence matrix and gray level size zone matrix features) contributed to the model construction. Figure 3 depicts expression heat maps for intratumoral first-order kurtosis by Laplacian of Gaussian (σ = 2 mm) and peritumoral gray level nonuniformity normalized without filters from representative patients. The heat maps highlighted the subtle tumoral heterogeneity that was hardly visible in original CT images. The baseline tumor volume was not predictive of pCR in this study (eFigure 2 in the Supplement). Calibration curve and decision curve also showed good calibration and clinical application performance of the model (eFigure 3 and eFigure 4 in the Supplement). Using the optimal cutoff value of 0.47, patients with radiomics scores greater than or equal to 0.47 were predicted to have pCR, and those with radiomics score less than 0.47 were predicted to not have pCR (eFigure 5 in the Supplement).

**Table 2.** Description of Selected Features in the Combined Model Using Support Vector Machine With Radial Basis Function Kernel

Group and filter^a^	Feature class	Feature
Intratumoral feature		
Wavelet (HHH)	GLSZM	Large area low gray level emphasis
LoG (σ = 2 mm)	First order	Kurtosis
Wavelet (LLL)	GLSZM	Large area low gray level emphasis
LoG (σ = 2 mm)	First order	Median
LoG (σ = 5 mm)	GLCM	Sum average
LoG (σ = 2 mm)	GLCM	Inverse variance
Wavelet (HLH)	First order	Median
Peritumoral feature		
Wavelet (HHH)	First order	Median
Wavelet (HHH)	GLCM	Inverse difference normalized
Wavelet (LLL)	GLCM	Cluster shade
LoG (σ = 1 mm)	First order	Kurtosis
Original	GLSZM	Gray level nonuniformity normalized
Wavelet (HLH)	First order	Kurtosis

Abbreviations: GLSZM, gray level size zone matrix; GLCM, gray level co-occurrence matrix; LoG, Laplacian of Gaussian.

^a^ For wavelet filtration, H and L represent high-pass filter and low-pass filter on the x, y, and z directions, respectively.

Except for the optimal intratumoral, peritumoral, and combined models, no clinical factors were significantly associated with pCR in univariable analysis (eTable 4 in the Supplement). We further stratified the prediction performance of the combined model by clinical factors. Interestingly, family history of cancer was significantly associated with the discriminative performance (eTable 5 in the Supplement). The AUC for the subgroup of patients with family history of cancer was 0.977 (95% CI, 0.944-1.000), compared with 0.868 (95% CI, 0.813-0.922) for patients with no family history of cancer (*P* < .001). The likelihood of different performance existed in subgroups stratified by tobacco or alcohol use, with no statistical significance.

### Radiogenomics Analysis

To elucidate the pathophysiological association with the radiomics signatures and how peritumoral radiomics features contributed to the predictive performance, we performed a radiogenomics analysis using RNA-seq data derived from pretreatment specimens from 40 patients in the training set. The range of the radiomics scores (0.353-0.613) in this subset basically overlapped with that of the original training set (0.347-0.613). Genes were ranked via correlation coefficients for the combined or intratumoral model, yielding 2 ranked gene lists for enrichment analysis. After simplifying the redundancy of enrichment results of the 2 models through an enrichment map (eFigure 6 in the Supplement), most of the enriched gene sets were indicated to be immune related. For both the combined and intratumoral models, clusters of interferon-γ, T cell–related immunity, B cell–related immunity, and multiple families of interleukin gene sets had high normalized enrichment scores (eTable 6 in the Supplement). Exclusive clusters that were associated with only the combined model, rather than the intratumoral model, might show the contribution of peritumoral features to the identification of pCR (eg, type I interferon, lymphocyte apoptosis, and natural killer cell) ranked by the highest normalized enrichment scores for gene set in the cluster. Because most of the gene sets enriched in the immune-related clusters had positive normalized enrichment scores, the corresponding phenotypes were positively associated with radiomics scores and nCRT response.

## Discussion

The pretreatment prediction of pCR is important for developing individualized therapy. Previous radiomics studies^20,21,22,23^ of patients with EC receiving nCRT mainly focused on intratumoral features alone and established models using data of small cohorts from single institutions without validating performance externally. There is emerging evidence that predictive models should not be limited to mere tumor areas. Recent studies^24,25,26,37,38,39,40^ have shown that the surrounding regions may provide complementary information on tumor heterogeneity in other cancers. Here we proposed a noninvasive, CT-based radiomics model with favorable predictive value using both intratumoral and peritumoral radiomics features to predict the possibility of pCR in patients with ESCC before receiving nCRT. Although a wide range of performance across different classifiers was achieved in the test set, the combination of intratumoral and peritumoral features always improved the classification accuracy regardless of the choice of classifier, confirming the additional predictive value of peritumoral radiomics features. We only included 1 external cohort for validation because of data resource restriction. More data sets are needed to further validate the optimal classifier for clinical practice.

The risk stratification analysis demonstrated that the prediction performance could vary in terms of family history of cancer, which may correlate with oncogenesis heterogeneity, reflecting the differing genetic traits. Previous studies on breast cancer found that risk stratification models could perform differently for patients with various family histories of tumor.^41,42^ Familial risks could reflect the shared genetic susceptibility and similar exposures to environmental risk factors and have been reported to be associated with esophageal carcinogenesis and prognosis.^43^ Patients could benefit from risk evaluation that involves collection of detailed information on clinical risk factors. Further clinical studies on large-scale data sets are needed to fully address this question.

Various lines of evidence have shown stroma-mediated and lymphocyte-related resistance of chemotherapy or radiotherapy in malignant neoplasms.^44,45,46,47,48,49^ Previous radiomics studies have associated the treatment response prediction power of intratumoral and peritumoral radiomics with immune microenvironment. Beig et al^37^ reported that the diagnostic value of perinodular radiomics could be linked to the dense distribution of tumor-infiltrating lymphocytes and tumor-associated macrophages surrounding the core area in lung adenocarcinoma. Braman and colleagues^38^ observed a significant correlation between peritumoral radiomic features immediately outside breast cancer and lymphocytic density. Jiang et al^50^ proposed a radiomics signature by using both intratumoral and peritumoral CT features to evaluate the ImmunoScore calculated by immune cell biomarkers in tumor core and invasive margin and proved it to be prognostic and predictive of chemotherapy response in gastric cancer. Our results demonstrate that the combined intratumoral and peritumoral radiomics model might be associated with lymphocyte-mediated immunity and cytokines such as interferons and interleukins, implying the significant predictive value and functions of microenvironmental immune components in the innate chemoresistance or radioresistance in ESCC. Wu and colleagues^51^ have investigated the molecular basis of imaging characteristics of tumor-adjacent parenchyma in breast cancer. The most enriched pathways were identical in the tumor and tumor-adjacent parenchyma gene profiles associated with the imaging characteristics and enriched genes in the 2 parts located upstream and downstream, respectively. This provided evidence of a molecular association between the intratumoral and peritumoral regions. In the present study, we used the intratumoral gene profiles to explore the associated peritumoral biological basis and found that the exclusive clusters associated with the combined model were also immune related (eg, type I interferon) and underlined the potentially important role of the surrounding stroma or peritumoral tissue in therapy resistance.

### Limitations

There are several limitations to the current study. First, generation of peritumoral shell expansion as the peritumoral area is not feasible because of overlaps of attenuation values between ROIs and excluded areas and possibly insufficient dilation covering the adjacent enlarged nodes. The contour of the peritumoral area relied on manual delineation by experienced radiologists, which was time-consuming and labor-intensive. Further study on automatic volumetric segmentation is required to simplify the process. Second, our test-retest analysis showed that peritumoral radiomics features were less robust than the intratumoral features (1208 of 1316 of intratumoral and 1036 of 1316 of the peritumoral extracted feature with intraclass correlation coefficients >0.80, shown in eTable 7 in the Supplement). A previous test-retest study^52^ using the RIDER data set investigated the reproducibility of intratumoral features over a wide range of imaging settings. To account for this variability, further prospective test-retest studies are needed to assess peritumoral feature robustness. Third, the RNA-seq data were representative only for the genetic profile of tumors. We did not perform RNA-seq on peritumoral tissue, so the actual pathophysiological process for the peritumoral tissue affecting chemoradiotherapy sensitivity remains unclear. Furthermore, the radiogenomics analysis was based on a small sample size (40 patients in the training set) because of the difficulty in assessing pretreatment samples in a retrospective setting, and we did not validate the radiogenomics results in the test set. Further studies with larger sample sizes are needed to confirm its role in therapy response.

## Conclusions

These findings suggest that peritumoral radiomics features provide valuable information about response to nCRT treatment. A combination of intratumoral and peritumoral radiomics features could enhance the predictive ability of radiomics model in identifying pCR in patients with ESCC. The microenvironmental immune components were most likely to be associated with both the intratumoral and peritumoral radiomics prediction.

## Notes

Supplement. **eAppendix 1. **Supplemental Methods **eReferences.** **eFigure 1.** Flowchart of Patient Selection **eFigure 2.** Stratification of the Combined Model by the Baseline Tumor Volume in Voxel Size **eFigure 3. **Calibration Curve of the Optimal Combined Radiomics Model **eFigure 4.** Decision Curve of the Optimal Combined Radiomics Model **eFigure 5. **Diagnostic Possibilities Calculated by the Combined Radiomics Model for Each Patient **eFigure 6. **Enrichment Map for Enriched Gene Sets Associated With the Combined Radiomics Model and the Intratumoral Model **eTable 1.** Comparison of Predictive Performance Across Different Classifiers **eTable 2.** Radiomics Features in the Intratumoral and Peritumoral Models **eTable 3. **Number of Selected Features Across Different Classifiers **eTable 4. **Univariable Analysis on Radiomics Models and Clinical Factors Associated for pCR **eTable 5. **Stratified Prediction Performance in Different Risk Subgroups **eTable 6. **Clusters of Gene Sets for the Enrichment Analysis **eTable 7. **Numbers of Selected Features Through the Construction Procedure of the Combined Radiomics Model **eAppendix 2.** Ranked Gene List
