# Supplementary material for: Assessment of Intratumoral and Peritumoral Computed Tomography Radiomics for Predicting Pathological Complete Response to Neoadjuvant Chemoradiation in Patients With Esophageal Squamous Cell Carcinoma
Source: JAMA Netw Open. 2020 Sep 10;3(9):e2015927. doi: 10.1001/jamanetworkopen.2020.15927 (PMC7489831; doi:10.1001/jamanetworkopen.2020.15927)
Supplement: Supplement. — eAppendix 1. Supplemental Methods eReferences. eFigure 1. Flowchart of Patient Selection eFigure 2. Stratification of the Combined Model by the Baseline Tumor Volume in Voxel Size eFigure 3. Calibration Curve of the Optimal Combined Radiomics Model eFigure 4. Decision Curve of the Optimal Combined Radiomics Model eFigure 5. Diagnostic Possibilities Calculated by the Combined Radiomics Model for Each Patient eFigure 6. Enrichment Map for Enriched Gene Sets Associated With the Combined Radiomics Model and the Intratumoral Model eTable 1. Comparison of Predictive Performance Across Different Classifiers eTable 2. Radiomics Features in the Intratumoral and Peritumoral Models eTable 3. Number of Selected Features Across Different Classifiers eTable 4. Univariable Analysis on Radiomics Models and Clinical Factors Associated for pCR eTable 5. Stratified Prediction Performance in Different Risk Subgroups eTable 6. Clusters of Gene Sets for the Enrichment Analysis eTable 7. Numbers of Selected Features Through the Construction Procedure of the Combined Radiomics Model eAppendix 2. Ranked Gene List [file jamanetwopen-e2015927-s001.pdf]

Supplementary Online Content

Hu Y, Xie C, Yang H, et al. Assessment of intratumoral and peritumoral computed tomography radiomics for predicting pathological complete response to neoadjuvant chemoradiation in patients with esophageal squamous cell carcinoma. *JAMA Netw Open*. 2020;3(9):e2015927. doi:10.1001/jamanetworkopen.2020.15927

eAppendix 1. Supplemental Methods
eReferences.
eFigure 1. Flowchart of Patient Selection
eFigure 2. Stratification of the Combined Model by the Baseline Tumor Volume in Voxel Size
eFigure 3. Calibration Curve of the Optimal Combined Radiomics Model
eFigure 4. Decision Curve of the Optimal Combined Radiomics Model
eFigure 5. Diagnostic Possibilities Calculated by the Combined Radiomics Model for Each Patient
eFigure 6. Enrichment Map for Enriched Gene Sets Associated With the Combined Radiomics Model and the Intratumoral Model
eTable 1. Comparison of Predictive Performance Across Different Classifiers
eTable 2. Radiomics Features in the Intratumoral and Peritumoral Models
eTable 3. Number of Selected Features Across Different Classifiers
eTable 4. Univariable Analysis on Radiomics Models and Clinical Factors Associated for pCR
eTable 5. Stratified Prediction Performance in Different Risk Subgroups
eTable 6. Clusters of Gene Sets for the Enrichment Analysis
eTable 7. Numbers of Selected Features Through the Construction Procedure of the Combined Radiomics Model
eAppendix 2. Ranked Gene List

This supplementary material has been provided by the authors to give readers additional information about their work.

© 2020 Hu Y et al. *JAMA Network Open.*

eAppendix 1. Supplemental Methods

1. Neoadjuvant chemoradiotherapy regimens

For institution 1, 75 mg/m^2 cisplatin on day 1 and 25 mg/m^2 vinorelbine on days 1 and 8 for 2 cycles, or 25 mg/m^2 cisplatin and 25 mg/m^2 docetaxel on days 1, 8, 15 and 21 were administrated intravenously, with a total dose of 40 or 44 Gy for concurrent radiotherapy. For institution 2, patients received 50 mg/m^2 paclitaxel and carboplatin AUC 2 for 5 cycles or 100 mg/m^2 cisplatin and 500 mg/m^2 fluorouracil for 4 days for weeks 1 and 5, with a total dose of 40 or 41.4 Gy for concurrent radiotherapy.

2. CT image acquisition

Parameter	Institution 1	Institution 2
Scanner	Aquilion TSX-101A (Toshiba Medical Systems, Tokyo, JP) or Discovery 750 HD (GE Healthcare, Milwaukee, WI)	Discovery VCT, GE Healthcare (GE Healthcare, Milwaukee, WI)
Dose of iodinated CT contrast agent	1.5 ml/kg	100 mL
Injection rate of iodinated CT contrast agent	2.5 ml/s	3 ml/s
Slice thickness (mm)	2.5 (median)	5 (median)
Tube voltage (kVp)	120	120
Tube current (mA)	200-400	200-300

3. Radiomics Feature extraction and definitions

The resampled voxel sizes were set to 1×1×5 mm³ voxels to standardize the slice thickness. Image intensities were binned by 25 HU and voxel array shift was set on 1000. Care was taken to threshold the Hounsfield units (HU) of the CT scan (-100~400 HU) in order to remove air and bone pixels. Original images depicted the baseline structural characteristics. Wavelet filtration (high and low pass filters) filtered original images directionally with x, y and z directions respectively, yielding 8 different combinations of decompositions. LoG filter, also called edge enhancement filter, convolved the image with the second derivative (Laplacian) of a Gaussian kernel. The Gaussian kernel is sensitive to areas with rapidly changing intensities and enhancing edges. The width of the filter in the Gaussian kernel is determined by σ and we adopted σ with the range of 1-5 mm at 1 mm interval to achieve both fine (low σ values) and coarse (high σ values) textures. These filtrations could capture more detailed disruptions in different orientations.

The features can be divided into 3 groups: (a) first-order statistics, describing commonly used and basic metrics for distributions of the voxel intensity within the ROI; (b) shape features, including descriptors of the two-dimensional and three-dimensional morphological properties (size and shape) of the ROI; and (c) second-order features, consisting of high-dimensional textual features quantifying the spatial distribution of pixel intensities in all three-dimensional directions, which takes the spatial distribution of each voxel and the neighboring voxels into consideration. Most features defined below were in accordance with feature definitions as described by the Imaging Biomarker Standardization Initiative (IBSI), which were available in a separate document by Zwanenburg et al. (2016).[1] Here, we show the description of the radiomic features used in our optimal combined model:

© 2020 Hu Y et al. *JAMA Network Open.*

2.1 First order features

First-order statistics describe the distribution of voxel intensities within the image region defined by the mask through commonly used and basic metrics.

Let:

- **X** be a set of Np voxels included in the ROI
- $\mathbf{P}(i)$ be the first order histogram with Ng discrete intensity levels, where Ng is the number of non-zero bins, equally spaced from 0 with a width defined in the bin width parameter
- $p(i)$ be the normalized first order histogram and equal to $\mathbf{P}(i)/Np$

1) Median

The median gray level intensity within the ROI.

2) Kurtosis

$$\textit{kurtosis} = \frac{\mu_4}{\sigma^4} = \frac{\frac{1}{N_p}\sum_{i=1}^{N_p}(\mathbf{X}(i)-\bar{X})^4}{\left(\frac{1}{N_p}\sum_{i=1}^{N_p}(\mathbf{X}(i)-\bar{X})^2\right)^2}$$

Where μ_4 is the 4th central moment.

Kurtosis is a measure of the ‘peakedness’ of the distribution of values in the image ROI. A higher kurtosis implies that the mass of the distribution is concentrated towards the tail(s) rather than towards the mean. A lower kurtosis implies the reverse: that the mass of the distribution is concentrated towards a spike near the Mean value.

2.2 Second order features

2.2.1 Gray Level Size Zone Matrix (GLSZM) Features

A Gray Level Size Zone (GLSZM) quantifies gray level zones in an image. A gray level zone is defined as the number of connected voxels that share the same gray level intensity. A voxel is considered connected if the distance is 1 according to the infinity norm (26-connected region in a 3D, 8-connected region in 2D). In a gray level size zone matrix $P(i,j)$ the $(i,j)^{th}$ element equals the number of zones with gray level i and size j appear in image. Contrary to GLCM and GLRLM, the GLSZM is rotation independent, with only one matrix calculated for all directions in the ROI.[2] As a two-dimensional example, consider the following 5x5 image, with 5 discrete gray levels:

$$\mathbf{I} = \begin{bmatrix} 5 & 2 & 5 & 4 & 4 \\ 3 & 3 & 3 & 1 & 3 \\ 2 & 1 & 1 & 1 & 3 \\ 4 & 2 & 2 & 2 & 3 \\ 3 & 5 & 3 & 3 & 2 \end{bmatrix}$$

The GLSZM then becomes:

© 2020 Hu Y et al. *JAMA Network Open.*

$$\mathbf{P} = \begin{bmatrix} 0 & 0 & 0 & 1 & 0 \\ 1 & 0 & 0 & 0 & 1 \\ 1 & 0 & 1 & 0 & 1 \\ 1 & 1 & 0 & 0 & 0 \\ 3 & 0 & 0 & 0 & 0 \end{bmatrix}$$

Let:

- Ng be the number of discrete intensity values in the image
- Ns be the number of discrete zone sizes in the image
- Np be the number of voxels in the image
- Nz be the number of zones in the ROI, which is equal to $\sum_{i=1}^{N_g}\sum_{j=1}^{N_s}\mathbf{P}(i,j)$ and $1 \le Nz \le Np$
- $\mathbf{P}(i,j)$ be the size zone matrix
- $p(i,j)$ be the normalized size zone matrix, defined as $p(i,j) = \frac{\mathbf{P}(i,j)}{Nz}$

1) Large Area Low Gray Level Emphasis (LALGLE)

$$LALGLE = \frac{\sum_{i=1}^{N_g}\sum_{j=1}^{N_s}\frac{\mathbf{P}(i,j)j^2}{i^2}}{N_z}$$

LALGLE measures the proportion in the image of the joint distribution of larger size zones with lower gray-level values.

2) Gray Level Non-Uniformity Normalized (GLNN)

$$GLNN = \frac{\sum_{i=1}^{N_g}\left(\sum_{j=1}^{N_s}\mathbf{P}(i,j)\right)^2}{N_z^2}$$

GLNN measures the variability of gray-level intensity values in the image, with a lower value indicating a greater similarity in intensity values. This is the normalized version of the GLN formula.

2.2.2 Gray Level Co-occurrence Matrix (GLCM) Features

A Gray Level Co-occurrence Matrix (GLCM)[3] of size $Ng \times Ng$ describes the second-order joint probability function of an image region constrained by the mask and is defined as $\mathbf{P}(i,j|\delta,\theta)$. The $(i,j)^{\text{th}}$ element of this matrix represents the number of times the combination of levels i and j occur in two pixels in the image, that are separated by a distance of δ pixels along angle θ. The distance δ from the center voxel is defined as the distance according to the infinity norm. For δ=1, this results in 2 neighbors for each of 13 angles in 3D (26-connectivity) and for δ=2 a 98-connectivity (49 unique angles).

As a two-dimensional example, let the following matrix **I** represent a 5x5 image, having 5 discrete grey levels:

$$\mathbf{I} = \begin{bmatrix} 1 & 2 & 5 & 2 & 3 \\ 3 & 2 & 1 & 3 & 1 \\ 1 & 3 & 5 & 5 & 2 \\ 1 & 1 & 1 & 1 & 2 \\ 1 & 2 & 4 & 3 & 5 \end{bmatrix}$$

For distance δ=1 (considering pixels with a distance of 1 pixel from each other) and angle θ=0° (horizontal plane, i.e. voxels to the left and right of the center voxel), the following symmetrical GLCM is obtained:

$$\mathbf{P} = \begin{bmatrix} 6 & 4 & 3 & 0 & 0 \\ 4 & 0 & 2 & 1 & 3 \\ 3 & 2 & 0 & 1 & 2 \\ 0 & 1 & 1 & 0 & 0 \\ 0 & 3 & 2 & 0 & 2 \end{bmatrix}$$

Let:

- ϵ be an arbitrarily small positive number ($\approx 2.2\times10^{-16}$)
- $\mathbf{P}(i,j)$ be the co-I matrix for an arbitrary δ and θ
- $p(i,j)$ be the normalized co-I matrix and equal to $\mathbf{P}(i,j)\sum\mathbf{P}(i,j)$
- Ng be the number of discrete intensity levels in the image
- $px(i)=\sum Ngj=1P(i,j)$ be the marginal row probabilities
- $py(j)=\sum Ngi=1P(i,j)$ be the marginal column probabilities
- σx be the standard deviation of px
- σy be the standard deviation of py

By default, the value of a feature is calculated on the GLCM for each angle separately, after which the mean of these values is returned.

1) Inverse Difference Moment (IDM)

$$IDM = \sum_{k=0}^{N_g-1} \frac{p_{x-y}(k)}{1+k^2}$$

IDM (a.k.a Homogeneity 2) is a measure of the local homogeneity of an image. IDM weights are the inverse of the Contrast weights (decreasing exponentially from the diagonal i=j in the GLCM).

2) Sum Average

$$sum\ average = \sum_{k=2}^{2N_g} p_{x+y}(k)k$$

Sum Average measures the relationship between occurrences of pairs with lower intensity values and occurrences of pairs with higher intensity values.

© 2020 Hu Y et al. *JAMA Network Open.*

3) Inverse Variance

$$\textit{inverse variance} = \sum_{k=1}^{N_g-1} \frac{p_{x-y}(k)}{k^2}$$

Note that k=0 is skipped, as this would result in a division by 0.

4) Cluster Shade

$$\textit{cluster shade} = \sum_{i=1}^{N_g} \sum_{j=1}^{N_g} \left(i + j - \mu_x - \mu_y\right)^3 p(i, j)$$

Cluster Shade is a measure of the skewness and uniformity of the GLCM. A higher cluster shade implies greater asymmetry about the mean.

4. RNA sequencing procedure

Total RNA was isolated using AllPrep DNA/RNA Mini Kit (Qiagen) from 40 frozen specimens acquired through endoscopic biopsies in institution 1. RNA purity and concentration were measured by NanoDrop 2000 Microvolume UV-Vis spectrophotometer (Thermo Scientific). RNA quality was estimated by RNA integrity number (RIN) on the 2100 Bioanalyzer (Agilent) and samples with RIN over 6.0 were ensured for library construction. We used the Epicentre Ribo-zero rRNA Removal Kit (Epicentre) to remove ribosomal RNA (rRNA), and proceeded to generate libraries by using the NEBNext Ultra Directional RNA Library Prep Kit for Illumina (New England Biolabs). The first-strand cDNA was synthesized using random primers and reverse transcriptase followed by the synthesis of the second-strand cDNA by DNA Polymerase I and Rnase H. Then, DNA fragments underwent adenylation of the 3' ends and ligation of sequencing adapters. After cDNA purification on the AMPure XP system (Beckman Coulter), the quality and quantity of libraries were assessed by the 2100 Bioanalyzer (Agilent). Finally, the cDNA libraries were placed on a cBot System (Illumina) for cluster generation and sequenced on the Illumina HiSeq 4000 Platform at the Novogene Bioinformatics Institute (Beijing,China) to generate 150 bp paired-end reads. Raw reads were stored in the FASTQ format. In order to gain clean data, reads that included adapters, contained high proportion of N for no base information, or had low quality were removed. Clean reads were aligned to the hg19 human genome using TopHat v2.1.1, and mapped reads were assembled for each sample by Cufflinks v2. Annotation was performed according to the RefSeq database for mRNA and the GENCODE v21 database for lncRNA. To assess expression level, read counts were normalized and calculated as fragments per kilobase of exon per million fragments (FPKM) by Cuffdiff v2.2.1.

5. R packages

Radiomics features were harmonized to reduce the multicenter effect caused by different scanner and protocol settings. According to the statistical distribution of the dataset, nonparametric form of the model was adopted in which ComBat determined the transformation for each feature separately using "sva" R package.[4] Feature robustness was tested by intraclass correlation coefficients (ICCs) using "irr" R package.[5] The AUC of the receiver operating characteristic (ROC) curve was calculated and compare by "pRCO" R package.[6] The raw genomic data was preprocessed (background correction, log2-transformation and quantile normalization) using the Bioconductor package "affy".[7]

© 2020 Hu Y et al. *JAMA Network Open.*

eReferences

1. Zwanenburg A, Leger S, Vallières M, Löck S. Image biomarker standardisation initiative. *J arXiv preprint arXiv:07003.* 2016.
2. Thibault G, Fertil B, Navarro C, et al. Shape and texture indexes application to cell nuclei classification. *J International Journal of Pattern Recognition Artificial Intelligence.* 2013;27(01):1357002.
3. Haralick RM, Shanmugam K, Dinstein IHJITos, man,, cybernetics. Textural features for image classification. 1973(6):610-621.
4. Leek JT, Johnson WE, Parker HS, Jaffe AE, Storey JD. The sva package for removing batch effects and other unwanted variation in high-throughput experiments. *J Bioinformatics.* 2012;28(6):882-883.
5. Gamer M, Lemon J, Fellows I, Singh PJRANhcr-powpiip. Package irr: Various Coefficients of Interrater Reliability and Agreement (versión 0.84). 2012.
6. Robin X, Turck N, Hainard A, et al. pROC: an open-source package for R and S+ to analyze and compare ROC curves. *BMC Bioinformatics.* 2011;12:77.
7. Gautier L, Cope L, Bolstad BM, Irizarry RA. affy—analysis of Affymetrix GeneChip data at the probe level. *J Bioinformatics.* 2004;20(3):307-315.

© 2020 Hu Y et al. *JAMA Network Open.*

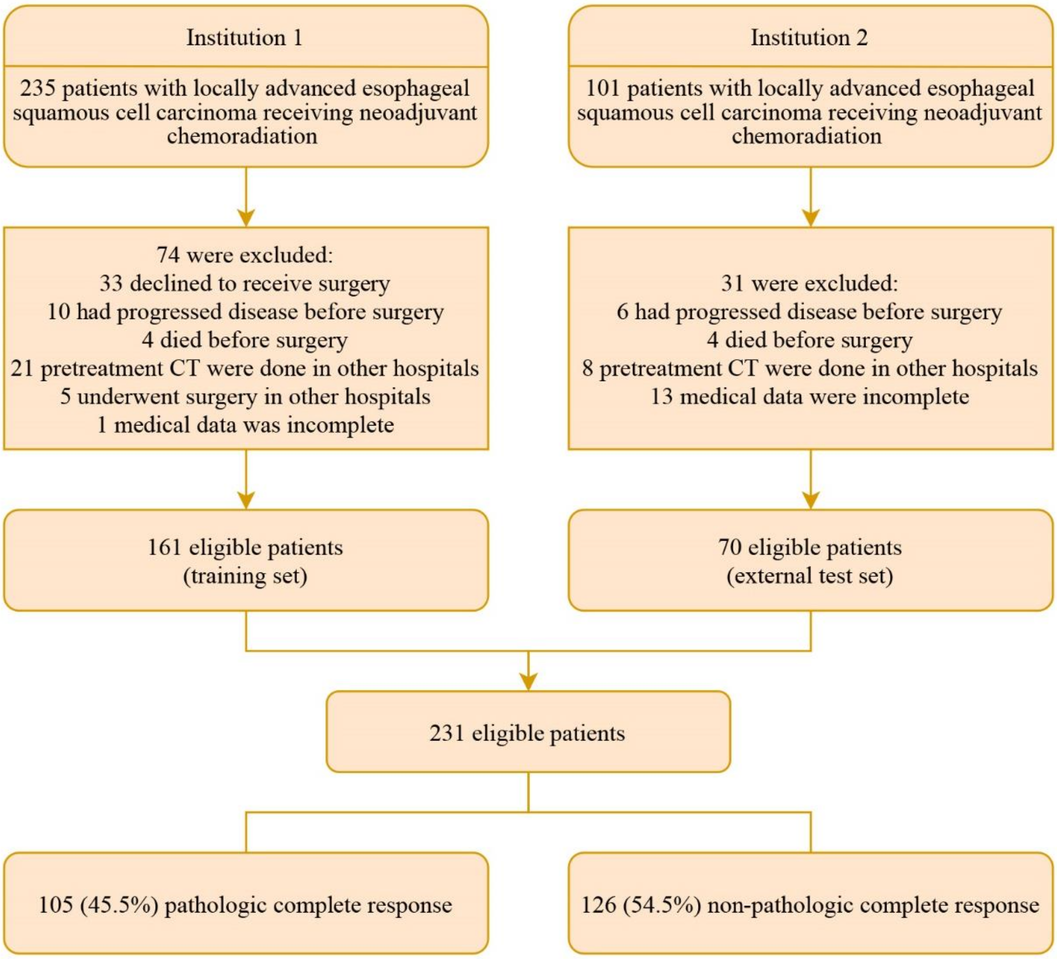

eFigure 1. Flowchart of Patient Selection.

© 2020 Hu Y et al. *JAMA Network Open.*

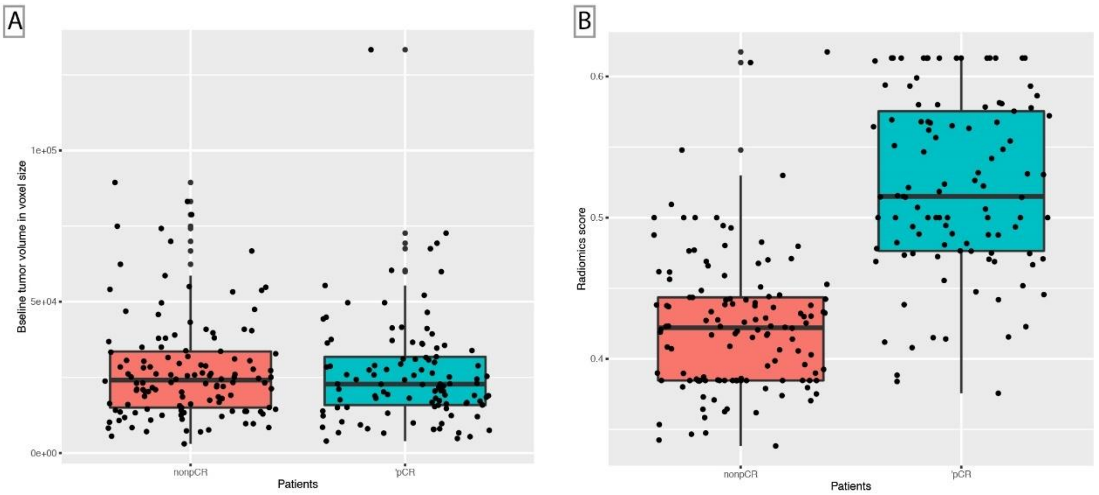

eFigure 2. Stratification of the Combined Model by the Baseline Tumor Volume in Voxel Size. Boxplots for pCR and non-pCR patients divided by (A) baseline tumor volume in voxel size (Univariable test, *P* =0.596) and (B) radiomics score (Univariable test, *P* < 0.001).

© 2020 Hu Y et al. *JAMA Network Open.*

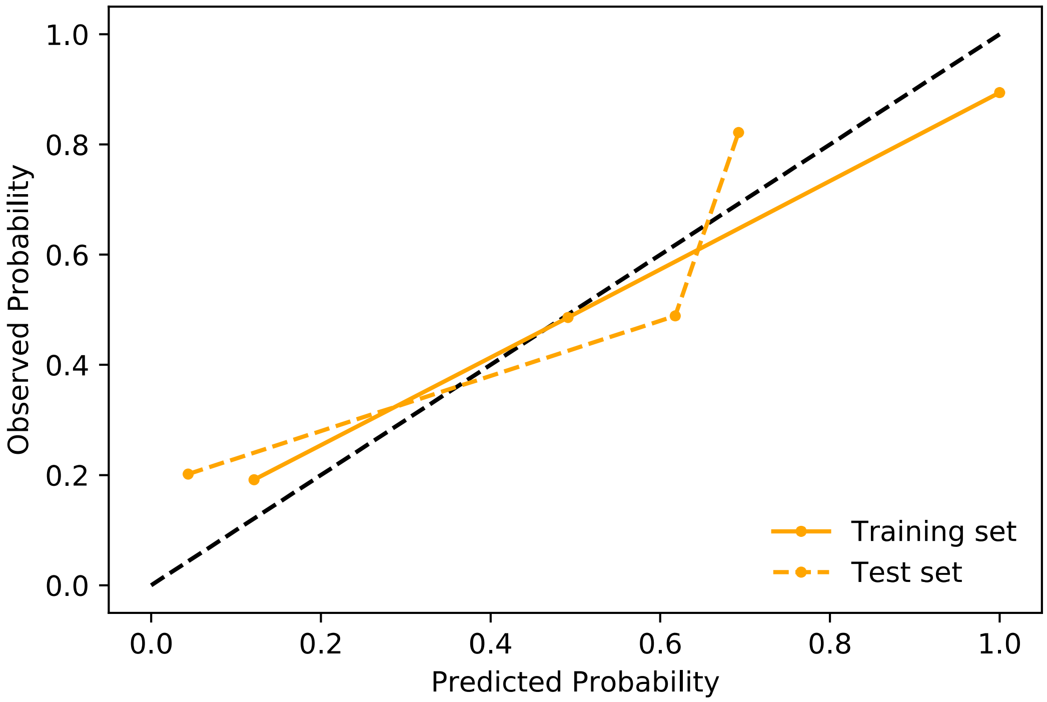

eFigure 3. Calibration Curve of the Optimal Combined Radiomics Model

© 2020 Hu Y et al. *JAMA Network Open.*

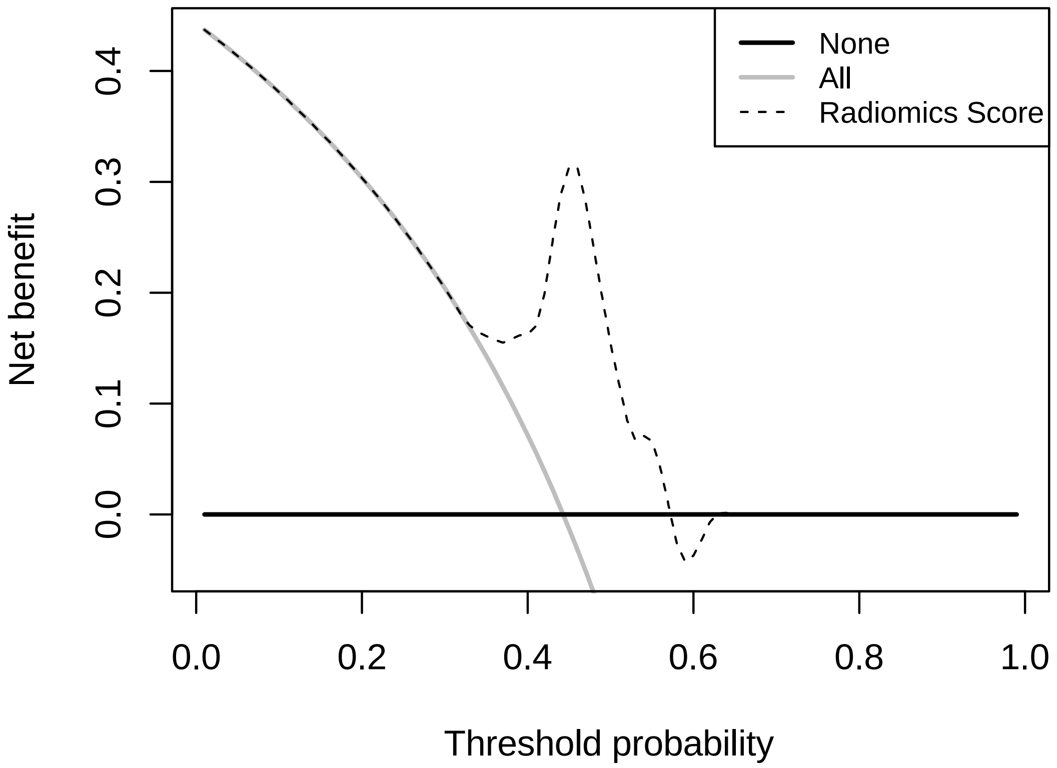

eFigure 4. Decision Curve of the Optimal Combined Radiomics Model

© 2020 Hu Y et al. *JAMA Network Open.*

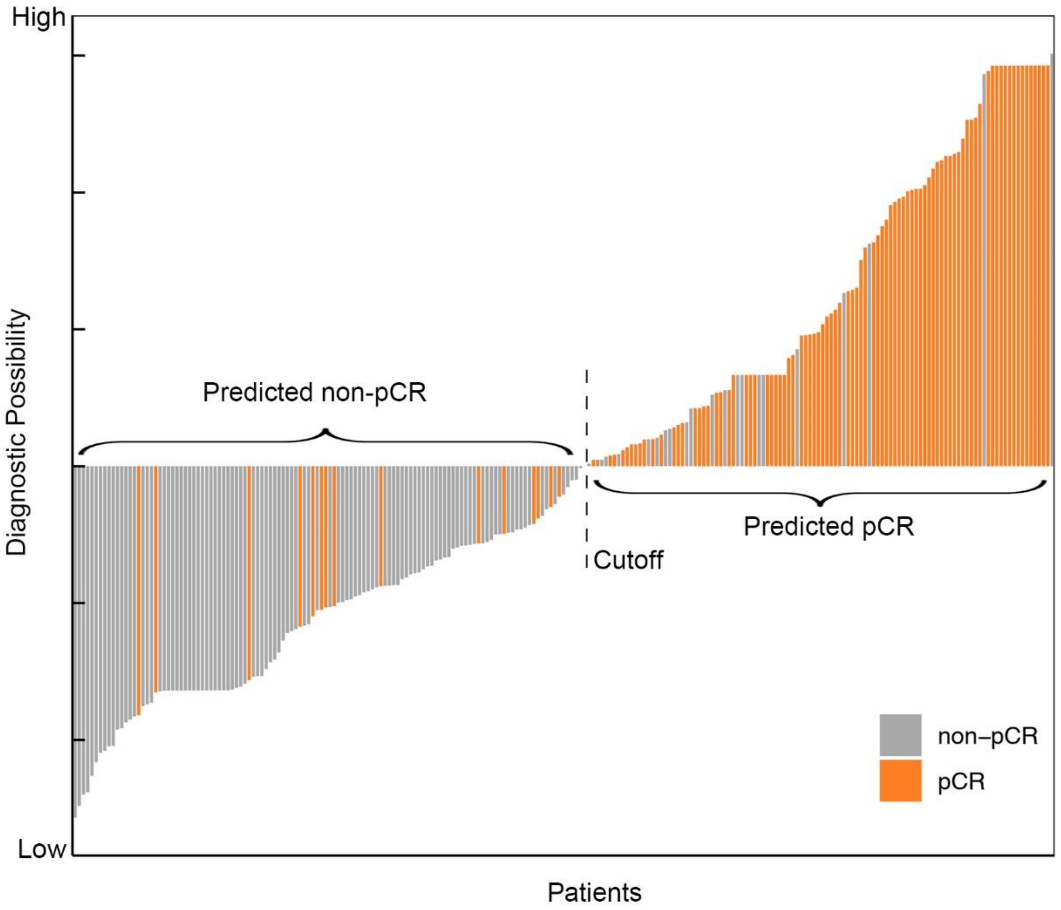

eFigure 5. Diagnostic Possibilities Calculated by the Combined Radiomics Model for Each Patient. Orange and gray bars show scores for pCR and non-pCR patients, respectively. pCR indicates pathologic complete response.

© 2020 Hu Y et al. *JAMA Network Open.*

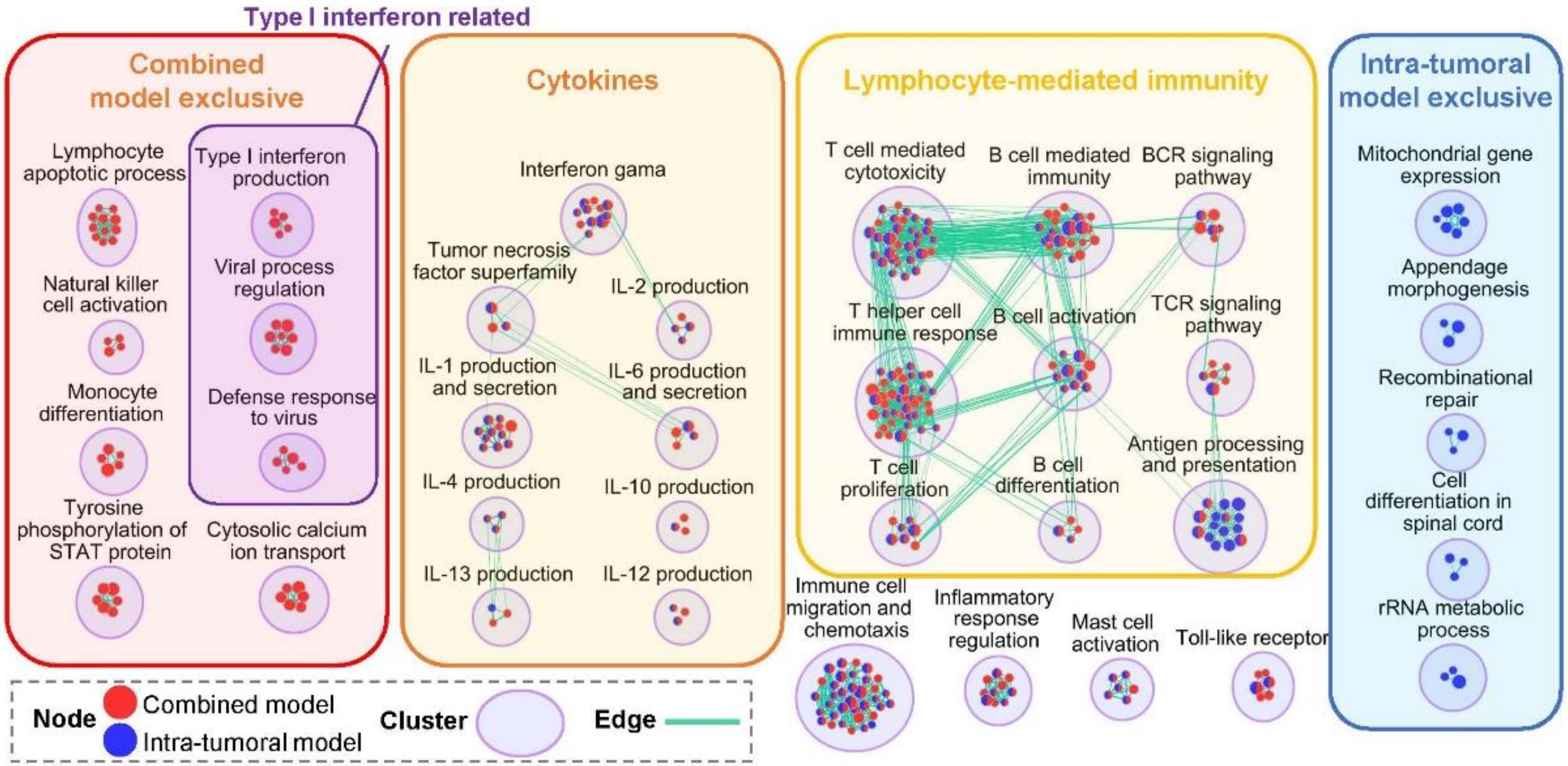

eFigure 6. Enrichment Map for Enriched Gene Sets Associated With the Combined Radiomics Model and the Intratumoral Model. Red and blue of node color represent enrichment in combined model and intra-tumoral model, respectively. Edges represent overlaps between gene sets. Clusters were generated using the AutoAnnotate application and manually modified and labeled.

© 2020 Hu Y et al. *JAMA Network Open.*

eTable 1. Comparison of Predictive Performance Across Different Classifiers

Classifier	AUC (95% CI)					
	Intra-tumoral model		Peri-tumoral model		Combined model	
	Training	Test	Training	Test	Training	Test
DC	1.000 (1.000-1.000)	0.724 (0.620-0.828)	1.000 (1.000-1.000)	0.688 (0.579-0.797)	1.000 (1.000-1.000)	0.740 (0.634-0.845)
KNN	0.769 (0.700-0.838)	0.680 (0.559-0.802)	0.847 (0.791-0.903)	0.675 (0.550-0.800)	0.777 (0.708-0.845)	0.746 (0.634-0.857)
Linear-SVM	0.729 (0.650-0.808)	0.660 (0.527-0.792)	0.809 (0.742-0.876)	0.673 (0.545-0.802)	0.812 (0.745-0.880)	0.749 (0.672-0.872)
LR	0.793 (0.724-0.862)	0.670 (0.535-0.805)	0.886 (0.833-0.939)	0.625 (0.490-0.759)	0.816 (0.752-0.880)	0.701 (0.576-0.827)
NB	0.787 (0.717-0.857)	0.700 (0.577-0.824)	0.816 (0.747-0.885)	0.634 (0.504-0.765)	0.900 (0.852-0.949)	0.800 (0.696-0.904)
RBF-SVM	0.881 (0.827-0.935)	0.730 (0.609-0.850)	0.895 (0.847-0.943)	0.726 (0.609-0.843)	0.906 (0.858-0.953)	0.852 (0.753-0.951)
RF	1.000 (0.999-1.000)	0.641 (0.512-0.770)	1.000 (0.9992-1.000)	0.676 (0.551-0.801)	1.000 (1.000-1.000)	0.725 (0.607-0.843)
XGboost	1.000 (1.000-1.000)	0.699 (0.571-0.827)	1.000 (1.000-1.000)	0.734 (0.613-0.854)	1.000 (1.000-1.000)	0.772 (0.652-0.891)

Abbreviations: AUC, area under the receiver operating characteristic curve; DC, decision tree; KNN, k-nearest neighbors; linear-SVM, support vector machine with linear kernel; LR, linear regression; NB, naive bayes; RBF-SVM, support vector machine with radial basis function kernel; RF, random forest; XGboost, extreme gradient boosting.

© 2020 Hu Y et al. *JAMA Network Open.*

eTable 2. Radiomics Features in the Intratumoral and Peritumoral Models

Model	Filter[a]	Feature class	Feature
Intra-tumoral model (RBF - SVM)	LoG (σ=1mm)	GLCM	Inverse Variance
	LoG (σ=2mm)	First order	Kurtosis
	LoG (σ=2mm)	GLCM	Inverse Variance
	LoG (σ=3mm)	First order	90 Percentile
	Original	GLRLM	Gray Level Variance
	Original	GLRLM	Run Entropy
	Original	NGTDM	Strength
	Wavelet (HHH)	GLSZM	Size Zone Non-Uniformity Normalized
	Wavelet (HHH)	GLSZM	Zone Variance
	Wavelet (HHL)	GLSZM	Size Zone Non-Uniformity Normalized
	Wavelet (HLH)	First order	Median
	Wavelet (HLH)	First order	Root Mean Squared
	Wavelet (HLL)	First order	90 Percentile
	Wavelet (LHH)	GLDM	Dependence Non-Uniformity
	Wavelet (LLH)	First order	Maximum
	Wavelet (LLH)	NGTDM	Strength
Peri-tumoral model (XGboost)	Wavelet (HHH)	First order	Kurtosis
	LoG (σ=4mm)	GLRLM	Run Percentage
	Wavelet (LHH)	GLRLM	Long Run Low Gray Level Emphasis
	Wavelet (LHL)	GLSZM	Large Area Low Gray Level Emphasis
	Original	GLCM	Inverse Variance
	Original	GLCM	Correlation
	Original	NGTDM	Busyness
	Original	First order	Variance

Abbreviations: RBF-SVM, support vector machine with radial basis function kernel, XGboost, extreme gradient boosting; LoG indicates Laplacian of Gaussian; GLCM, Gray Level Co-occurrence Matrix; GLRLM, Gray Level Run Length Matrix; NGTDM, Neighboring Gray Tone Difference Matrix; GLSZM, Gray Level Size Zone Matrix; GLDM, Gray Level Dependence Matrix; MCC, Maximal Correlation Coefficient.

[a] For wavelet filtration, “H” and “L” represent high pass filter and low pass filter on the x, y, z directions.

© 2020 Hu Y et al. *JAMA Network Open.*

eTable 3. Number of Selected Features Across Different Classifiers

Classifier	Intra-tumoral model	Peri-tumoral model	Combined model
DC	6	4	6
KNN	8	12	13
Linear-SVM	13	17	17
LR	18	13	14
NB	15	14	13
RBF-SVM	16	9	13
RF	5	7	13
XGboost	7	8	14

Abbreviations: DC, decision tree; KNN, k-nearest neighbors; linear-SVM, support vector machine with linear kernel; LR, linear regression; NB, naive bayes; RBF-SVM, support vector machine with radial basis function kernel; RF, random forest; XGboost. extreme gradient boosting.

© 2020 Hu Y et al. *JAMA Network Open.*

eTable 4. Univariable Analysis on Radiomics Models and Clinical Factors Associated for pCR

**Parameter**	**Unadjusted OR (95% CI)**	***P* value**
Intra-tumoral model		
Predicted non-pCR	1.00	-
Predicted pCR	27.81 (14.06, 58.33)	<.001
Peri-tumoral model		
Predicted non-pCR	1.00	-
Predicted pCR	12.28 (6.69, 23.36)	<.001
Combined model		
Predicted non-pCR	1.00	-
Predicted pCR	57.37 (25.07, 150.98)	<.001
Sex		
Male	1.00	-
Female	1.03 (0.51,2.06)	.92
Age	0.99 (0.96,1.02)	.36
Clinical T stage[a]		
T1b+T2	1.00	-
T3+T4a	0.89 (0.46, 1.70)	.72
Clinical N stage		
N0+N1	1.00	-
N2+N3	0.62 (0.36, 1.04)	.07
Clinical stage group		
I+II	1.00	-
III+IV A	0.81 (0.38, 1.72)	.99
Tumor location		
Proximal third	1.00	-
Middle third	0.58 (0.21, 1.53)	.28
Distal third	0.58 (0.20, 1.56)	.28
Histologic Grade		
1	1.00	-
2	0.72 (0.20, 2.49)	.60
3	0.60 (0.16, 2.18)	.43
Smoking status		
No	1.00	-
Yes	0.90 (0.53, 1.53)	.69
Drinking status		
No	1.00	-
Yes	0.70 (0.41, 1.19)	.19
Family history of cancer		
No	1.00	-
Yes	1.13 (0.59, 2.15)	.72

© 2020 Hu Y et al. *JAMA Network Open.*

Abbreviations: pCR, pathologic complete response; OR, odds ratio.

[a] The American Joint Committee on Cancer TNM staging system (8th edition).

© 2020 Hu Y et al. *JAMA Network Open.*

eTable 5. Stratified Prediction Performance in Different Risk Subgroups

Characteristic	No. of patients (%)	AUC (95% CI)	*P* value[a]
Sex			
Male	192 (83.1)	0.884 (0.833-0.934)	-
Female	39 (16.9)	0.922 (0.841-1.000)	.436
Clinical T stage[b]			
T1b+T2	46 (19.9)	0.885 (0.783-0.986)	-
T3+T4a	185 (80.1)	0.890 (0.840-0.941)	.920
Clinical N stage			
N0+N1	119 (51.5)	0.897 (0.840-0.955)	-
N2+N3	112 (48.5)	0.871 (0.797-0.945)	.579
Clinical stage group			
I+II	32 (13.9)	0.828 (0.674-0.982)	-
III+IV A	199 (86.1)	0.896 (0.849-0.943)	.415
Tumor location			
Proximal third	19 (8.2)	0.909 (0.780-1.000)	-
Middle third	126 (54.5)	0.894 (0.834-0.954)	.839
Distal third	86 (37.3)	0.877 (0.798-0.957)	.684
Histologic grade			
1	11 (4.8)	0.867 (0.636-1.000)	-
2	153 (66.2)	0.884 (0.827-0.940)	.891
3	67 (29.0)	0.901 (0.819-0.984)	.788
Smoking status			
No	87 (37.7)	0.933 (0.876-0.989)	-
Yes	144 (62.3)	0.859 (0.794-0.923)	.090
Drinking status			
No	139 (60.2)	0.929 (0.888-0.969)	-
Yes	92 (39.8)	0.833 (0.740-0.925)	.065
Family history of cancer			
No	185 (80.1)	0.868 (0.813-0.922)	-
Yes	46 (19.9)	0.977 (0.944-1.000)	<.001

Abbreviation: AUC, area under the receiver operating characteristic curve.

[a] *P* values were calculated by Delong test.

[b] The American Joint Committee on Cancer TNM staging system (8th edition).

© 2020 Hu Y et al. *JAMA Network Open.*

eTable 6. Clusters of Gene Sets for the Enrichment Analysis

Index	Clusters	Maximum NES[a]
	Combined and intra-tumoral models associated	
1	Interferon gamma	2.8607
2	T cell proliferation	2.6024
3	T cell mediated cytotoxicity	2.5895
4	T helper cell immune response	2.5409
5	B cell activation	2.4969
6	BCR signaling pathway	2.4711
7	immune cell migration and chemotaxis	2.3976
8	TCR signaling pathway	2.3804
9	B cell differentiation	2.3777
10	IL-4 production	2.3424
11	B cell mediated immunity	2.3274
12	mast cell activation	2.2924
13	Inflammatory response regulation	2.2648
14	IL-2 production	2.26
15	Antigen processing and presentation	2.217
16	IL-1 production and secretion	2.2105
17	IL-10 production	2.1619
18	IL-6 production and secretion	2.159
19	Tumor necrosis factor superfamily	2.1393
20	IL-12 production	2.0523
21	Toll-like receptor	1.8927
22	IL-13 production	1.8136
	Combined model associated only	
1	Type I interferon production	2.6705
2	lymphocyte apoptotic process	2.2512
3	Viral process regulation	2.2148
4	Defense response to virus	2.0846
5	Natural killer cell activation	2.0557
6	Tyrosine phosphorylation of STAT protein	1.9987
7	Cytosolic calcium ion transport	1.9689
8	Monocyte differentiation	1.8008

Abbreviation: NES indicates normalized enrichment score.

[a] The NES of the gene set with the maximum value in a cluster.

© 2020 Hu Y et al. *JAMA Network Open.*

eTable 7. Numbers of Selected Features Through the Construction Procedure of the Combined Radiomics Model

**Feature selection**	**Intra-tumoral features**							**Peri-tumoral features**						
	**Shape**	**Original**		**Wavelet filtration**		**LoG filtration**		**Shape**	**Original**		**Wavelet filtration**		**LoG filtration**	
		**First order**	**Second order**	**First order**	**Second order**	**First order**	**Second order**		**First order**	**Second order**	**First order**	**Second order**	**First order**	**Second order**
Initial number	14	18	75	144	600	90	375	14	18	75	144	600	90	375
Feature robustness	12	17	69	142	536	87	345	9	16	63	119	464	76	289
Filtration on correlated features	5	6	18	44	119	23	55	5	12	22	71	136	21	64
RFA	0	0	0	1	2	2	2	0	0	1	2	2	1	0

Abbreviations: LoG indicates Laplacian of Gaussian; RFA, Recursive feature addition.

© 2020 Hu Y et al. *JAMA Network Open.*

eAppendix 2. Ranked Gene List

https://github.com/chenyixie123/ESCC_spatial-heterogeneity

© 2020 Hu Y et al. *JAMA Network Open.*
